# Inner beauty supplement label information cues, consumer trust, and purchase intentions: cross-sectional evidence from South Korean consumers

**DOI:** 10.3389/fpsyg.2026.1864122

**Published:** 2026-08-17

**Authors:** Eunjeong Park, Ki Han Kwon

**Affiliations:** 1 Division of Beauty Arts Care, Department of Practical Arts, Graduate School of Culture and Arts, Dongguk University, Seoul, Republic of Korea; 2 College of General Education, Kookmin University, Seoul, Republic of Korea

**Keywords:** consumer trust, elaboration likelihood model, health supplement labels, inner beauty supplements, label information cues

## Abstract

**Introduction:**

Health supplement labels serve as a critical psychological interface through which consumers process health-relevant information and form trust judgments. This cross-sectional study examines how perceived label information cues are associated with consumer trust and self-reported purchase intentions among inner beauty supplement consumers in South Korea. Drawing on the Elaboration Likelihood Model (ELM) as an information-processing framework and Signaling Theory as a framework for perceived credibility, the study focuses on five label cue types: functional emphasis, outcome-focused expression, scientific evidence expression, emotional expression, and visual label credibility.

**Methods:**

A quantitative survey was administered to 502 consumers who had purchased and consumed inner beauty supplements within the preceding 6 months. Data were analyzed using exploratory factor analysis, confirmatory factor analysis, multiple regression, and both separate and integrated bootstrapped indirect association analyses.

**Results:**

The CFA results supported the seven-factor measurement model, and additional validity diagnostics supported construct reliability, convergent validity, and discriminant validity. All five label cue types were positively associated with consumer trust. Functional emphasis, scientific evidence expression, emotional expression, and visual label credibility were also uniquely associated with purchase intention in the multiple regression model, whereas outcome-focused expression was not. Separate bootstrapped indirect association models showed significant indirect associations through consumer trust for all five cue types. In the integrated model, functional emphasis, scientific evidence expression, and emotional expression retained significant indirect associations through consumer trust, whereas outcome-focused expression and visual label credibility did not.

**Discussion:**

These indirect association findings should be interpreted as statistical indirect associations rather than as evidence of causal pathways, mechanisms, or exclusive indirect effects. Overall, the findings provide cross-sectional evidence that perceived label cues, product credibility, and purchase intentions are closely related in the inner beauty supplement context.

## 1 Introduction

Health psychology has long recognized that psychological processes including health information processing, trust formation, and attitude behavior relationships are central determinants of health-related decision making (Conner and Norman, 2005). These processes increasingly operate in the consumer marketplace, where individuals must evaluate health product labels to form credibility judgments before committing to a health-related purchase (Hung et al., 2017). Inner beauty supplements, which are positioned as functional-food or nutraceutical products targeting skin radiance, hair and nail health, and body composition through ingredients such as collagen peptides, hyaluronic acid, and antioxidants, represent an emerging and theoretically understudied context for health psychology research (Al Hajj et al., 2024; Lohani et al., 2024).

The global functional food market was valued at $168 billion in 2013 and is projected to reach $275 billion by 2025 (Contini et al., 2023), with South Korea representing a particularly advanced market for inner beauty innovation (Lohani et al., 2024). In South Korea, health functional foods are also governed by a regulatory framework that emphasizes scientific evidence for functional claims (Oh, 2025). Product labels constitute a key channel through which consumers encounter and evaluate health-relevant information prior to purchase (Siro et al., 2008; Annunziata and Vecchio, 2011). More broadly, labeling research has shown that label information and label types can influence consumer evaluations and purchase-related responses across food and cosmetic product contexts (Yang et al., 2021; Calderon-Monge et al., 2024; Do and Le, 2025). Prior research suggests that trust in product efficacy and perceived credibility are closely related to consumer responses to health information and health-related purchase intention (Siegrist et al., 2008; Stancu et al., 2017; Yang et al., 2024). Recent front-of-package label research further shows that consumer responses to health-oriented labels depend not only on the presence of health claims but also on the credibility, format, and explanatory information attached to those claims. Liu et al. (2025a) found that health labels alone may be discounted by consumers when they generate skepticism or perceived trade-offs, whereas credible information regarding FDA qualification can mitigate such negative responses. In a related study, Liu et al. (2025b) showed that consumers evaluate alternative FDA-endorsed “Healthy” label designs differently, highlighting the importance of label format and visual communication in shaping consumer preferences. These findings support the need to examine how different label cue types are associated with perceived credibility and purchase intention in health-related product categories.

From a health psychology perspective, product labels can be understood as health communication artifacts through which consumers encounter health claims, evaluate credibility, and form purchase intentions. Despite the centrality of trust formation in health psychology models of health behavior (Mayer et al., 1995; McKnight et al., 2002), the ways in which different label information cue types are associated with consumer trust and subsequent purchase intention remain theoretically underspecified and empirically underexamined in the inner beauty supplement domain.

This study addresses these gaps by developing and testing an ELM-informed (Petty and Cacioppo, 1986) and signaling-theory-based framework (Spence, 1973; Erdem and Swait, 1998) to examine how five empirically derived label cue types are associated with consumer trust and purchase intention among inner beauty supplement consumers in South Korea. Rather than directly testing ELM processing routes or causal trust transfer, the study uses ELM as an interpretive framework for classifying label cue types and Signaling Theory as a framework for understanding perceived product credibility under information asymmetry. The study makes four primary theoretical contributions: (1) it develops a five-dimensional label information cue taxonomy for inner beauty supplements; (2) it examines whether different label cue perceptions are differentially associated with consumer trust and purchase intention from an ELM-informed perspective; (3) it clarifies the role of consumer trust as a perceived product-credibility-related indirect association linking label cue perceptions and purchase intention; and (4) it compares separate and integrated indirect association models to distinguish cue-specific indirect associations from indirect associations that remain after accounting for shared variance among label cue types.

## 2 Theoretical background and hypotheses

### 2.1 Elaboration likelihood model and label information processing

The Elaboration Likelihood Model (ELM) (Petty and Cacioppo, 1986) identifies two broad routes through which consumers may process persuasive communications (El Hedhli and Zourrig, 2023). ELM has also been applied across a range of contemporary consumer-behavior contexts, including electronic word-of-mouth, advertising, and influencer-mediated communication (Shahab et al., 2021; Moradi and Zihagh, 2022; Pan, 2024; Park et al., 2024). The central route refers to relatively effortful processing in which individuals carefully consider the quality and relevance of information, whereas the peripheral route refers to lower-effort processing in which judgments are influenced by heuristic cues such as visual appeal, affective impressions, or source-related signals (Li and See-To, 2024). These routes are shaped by consumers’ motivation and ability to process information (Chaiken, 1980). In the present study, ELM is used as an interpretive framework for classifying label information cue types, rather than as a directly tested model of cognitive processing. In the context of health-related food and supplement labels, consumers may respond differently to information cues depending on their specificity, evidential quality, emotional appeal, and visual presentation. Prior research has shown that statistical health claims, which resemble information-rich cues, may be more persuasive than narrative claims in food product evaluation, and that individual differences in health knowledge can moderate these responses (Lin and Lee, 2021). Building on this logic, the present study treats functional emphasis expressions and scientific evidence expressions as information-rich cues, while emotional expressions and visual label credibility are treated as affective or heuristic cues. Outcome-focused expressions may combine goal-relevant information with heuristic appeal because they present visible or expected product outcomes. However, the present survey did not measure involvement, motivation, ability, health knowledge, cognitive elaboration, or argument scrutiny. Therefore, the findings should be interpreted as ELM-informed associations rather than as direct evidence of central- or peripheral-route processing.

### 2.2 Signaling theory and the quality signal function of label cues

In information asymmetry situations in which the seller has more knowledge about product quality than the buyer, Signaling Theory (Spence, 1973) explains how quality can be credibly communicated through observable signals. Erdem and Swait (1998) extended this theory to consumer markets by showing how brand signals lower consumers’ search costs and perceived risk, facilitating purchase intention.

Inner beauty supplements represent a product category in which information asymmetry is pronounced, as consumers cannot directly verify ingredient bioavailability or clinical efficacy prior to purchase (Nemade et al., 2025). In such settings, product labels serve as multidimensional signals proxying unobservable product quality characteristics (Zhu and Song, 2025). Scientific evidence expressions including clinical research citations, ingredient concentration data, and authorized certification marks serve as credible signals as long as the reputational costs for fraudulent use are high. Signaling Theory suggests that these cues may be positively associated with consumer trust and purchase intention because they can be perceived as credibility-related signals under information asymmetry.

### 2.3 Consumer trust as a product-credibility judgment

Consumer trust refers to consumers’ confidence in a product’s competence, reliability, and integrity (Mayer et al., 1995; McKnight et al., 2002). In health-related supplement contexts, trust is especially important because consumers cannot directly verify ingredient bioavailability, physiological efficacy, or long-term outcomes before purchase. In such information-asymmetric situations, consumers may use label information cues as credibility-related signals when forming judgments about the product (Spence, 1973; Erdem and Swait, 1998; Connelly et al., 2011). Although Trust Transfer Theory (Stewart, 2003) is relevant when trust in one source or context is transferred to another target, the present study did not identify a specific external trusted source from which trust was transferred to the product. Therefore, Trust Transfer Theory is treated only as a related conceptual perspective rather than as a directly tested theoretical mechanism. In this study, consumer trust is conceptualized more narrowly as perceived product credibility associated with label information cues.

Accordingly, the statistical indirect role of consumer trust is interpreted as a perceived-credibility-related association. Consumers who perceive label information cues as clear, credible, evidence-based, emotionally appealing, or professionally presented may also report higher product credibility, which in turn is associated with stronger purchase intention (Mayer et al., 1995; McKnight et al., 2002; Siegrist et al., 2008; Macready et al., 2020). This interpretation is consistent with Signaling Theory, which explains how observable cues may reduce uncertainty and serve as credibility signals in information-asymmetric markets (Spence, 1973; Erdem and Swait, 1998; Connelly et al., 2011), and with health psychology and food-consumer research emphasizing trust as a proximal correlate of health-related behavioral intention (Siegrist et al., 2008; Macready et al., 2020). However, because the present study did not identify a specific external trusted source, these findings should not be interpreted as evidence of causal trust transfer.

### 2.4 Inner beauty label information Cue typology

Drawing on ELM as an interpretive cue-classification framework (Petty and Cacioppo, 1986; Chaiken, 1980) and Signaling Theory as a perceived-credibility framework under information asymmetry (Spence, 1973; Erdem and Swait, 1998), this study defines five information cue types related to inner beauty supplement labels. Functional emphasis expressions refer to label statements that describe ingredient functions or skin-related benefits, such as statements about collagen synthesis or skin elasticity, consistent with prior research on health claims and functional-food information processing (Lähteenmäki, 2013; Hung et al., 2017). Outcome-focused expressions refer to label statements that emphasize visible or expected product outcomes, such as improvement in skin radiance after a specified period of use, which is relevant to how consumers interpret product benefits and health-related claims (Verbeke, 2005). Scientific evidence expressions include information such as clinical testing, ingredient concentration, research-based claims, or certification-related information, which may be perceived as credibility-related signals when consumers cannot directly verify product quality before purchase (Spence, 1973; Erdem and Swait, 1998). Emotional expressions refer to affective or aspirational wording that evokes beauty-related feelings, sensory imagery, or positive self-perceptions, consistent with research on affective and heuristic cues in persuasion (Chaiken, 1980). Visual label credibility refers to perceived professionalism, consistency, clarity, and credibility of visual label elements, including design, color, icons, and certification marks, which prior research has linked to trustworthiness and consumer responses to label design (Alberts and Van der Geest, 2011; Liu et al., 2025b). These categories are treated as perceived label cue dimensions rather than as direct measures of cognitive elaboration, central-route processing, peripheral-route processing, or trust transfer.

### 2.5 Hypotheses

#### 2.5.1 The role of label cues in consumer trust

Based on the ELM-informed cue classification and Signaling Theory, consumers who perceive label information cues as clear, credible, evidence-based, emotionally appealing, or professionally presented may report higher consumer trust, conceptualized here as perceived product credibility.

*H1a*: Functional emphasis expression is positively associated with consumer trust in inner beauty supplement products.

*H1b*: Outcome-focused expression is positively associated with consumer trust in inner beauty supplement products.

*H1c*: Scientific evidence expression is positively associated with consumer trust in inner beauty supplement products.

*H1d*: Emotional expression is positively associated with consumer trust in inner beauty supplement products.

*H1e*: Visual label credibility is positively associated with consumer trust toward inner beauty supplement products.

#### 2.5.2 Label cueing and purchase intention

In addition to their association with consumer trust, perceived label information cues may be associated with purchase intention because they provide information that consumers can use when evaluating inner beauty supplement products (Macready et al., 2020; Dolgopolova et al., 2015) Previous functional-food research likewise indicates that consumer purchase and use decisions are associated with health-related attitudes, perceived benefits, social and psychological motives, and product evaluations (Urala and Lähteenmäki, 2004; Verbeke, 2006; Carrillo et al., 2013; Barauskaite et al., 2018; Huang et al., 2019). Affective evaluations may also contribute to behavioral intentions, providing additional theoretical relevance for examining emotional label expressions in relation to purchase intention (Ajzen and Sheikh, 2013).

*H2a*: Functional emphasis expression is positively associated with purchase intention.

*H2b*: Outcome-focused expression is positively associated with purchase intention.

*H2c*: Scientific evidence expression is positively associated with purchase intention.

*H2d*: Emotional expression is positively associated with purchase intention.

*H2e*: Visual label credibility is positively associated with purchase intention.

#### 2.5.3 Consumer trust and purchase intention

Consumer trust has been consistently associated with purchase intention across consumer and health-related product contexts (McKnight et al., 2002; Pavlou and Fygenson, 2006; Siegrist et al., 2008; Wang et al., 2019; Macready et al., 2020). In health-related and high perceived-risk product categories, trust may serve as a proximal credibility judgment that is closely related to purchase intention (Siegrist et al., 2008; Macready et al., 2020).

*H3*: Consumer trust is positively associated with purchase intention toward inner beauty supplement products.

#### 2.5.4 The statistical indirect role of consumer trust

Building on Signaling Theory and the concept of perceived product credibility, this study proposes that label information cue perceptions are indirectly associated with purchase intention through consumer trust (Ares et al., 2008; Siegrist et al., 2008). This is especially expected for outcome-focused expressions, where consumers interpret result claims as credibility cues (Verbeke, 2005).

*H4a*: Consumer trust is expected to show a significant statistical indirect association between functional emphasis expression and purchase intention.

*H4b*: Consumer trust is expected to show a significant statistical indirect association between outcome-focused expression and purchase intention.

*H4c*: Consumer trust is expected to show a significant statistical indirect association between scientific evidence expression and purchase intention.

*H4d*: Consumer trust is expected to show a significant statistical indirect association between emotional expression and purchase intention.

*H4e*: Consumer trust is expected to show a significant statistical indirect association between visual label credibility and purchase intention.

**Figure 1:**
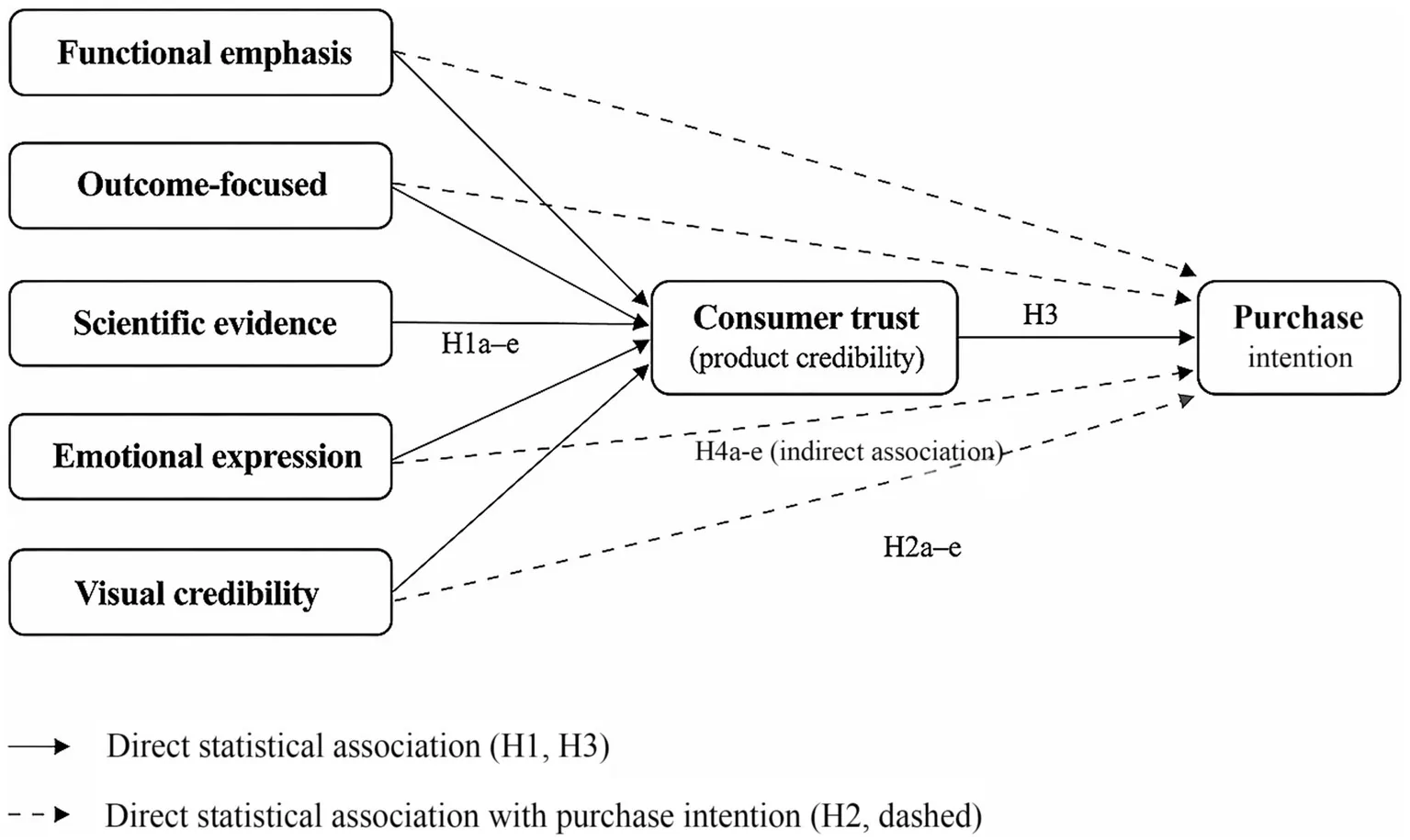
Conceptual research model.

The five conceived relationships linking the label information cue types and consumer trust to purchase intention constitute the conceptual research model (Figure 1).

Conceptual model illustrating the hypothesized statistical associations between five inner beauty supplement label information cue types (functional emphasis expression, outcome-focused expression, scientific evidence expression, emotional expression, and visual label credibility), consumer trust (perceived product credibility) and health-related purchase intention. Solid arrows represent hypothesized direct statistical associations; dashed arrows represent hypothesized statistical indirect associations through consumer trust. These arrows do not imply causal pathways because the study used a cross-sectional survey design. Hypotheses H1a–H1e represent label cue → consumer trust associations; H2a–H2e represent label cue → purchase intention associations; H3 represents consumer trust → purchase intention association; and H4a–H4e represent hypothesized statistical indirect associations through consumer trust.

## 3 Materials and methods

### 3.1 Research design and participants

This was a quantitative cross-sectional survey study. The target population comprised adults who had purchased and consumed inner beauty supplement products within the preceding 6 months. Data were collected through a professional online survey platform between January 15 and January 20, 2026. After removing participants who failed attention checks, a final valid sample of 502 individuals was retained. Respondents were not shown a standardized product label stimulus during the survey. Instead, they evaluated their general perceptions of label information cues based on inner beauty supplement products they had purchased or consumed within the preceding 6 months. Thus, the study captures general label-perception associations rather than responses to a single controlled label design. The sociodemographic characteristics of the sample are presented in Table 1. Women accounted for 426 participants (84.9%). The largest age group was the 30s (*n* = 277, 55.2%), followed by the 20s (*n* = 126, 25.1%) and ≥40 years (*n* = 99, 19.7%). The largest occupational group comprised company employees (68.7%), and most participants held bachelor’s degrees (71.7%). Monthly household income was concentrated in the KRW 3–4 million range (43.0%) and KRW 2–3 million range (30.3%), followed by < KRW 1 million (11.2%), ≥ KRW 4 million (8.8%), and KRW 1–2 million (6.8%). With respect to inner beauty supplement consumption patterns, skin health was the most commonly reported rationale for use (*n* = 239, 47.6%), followed by weight management (*n* = 116, 23.1%), hair and nail health (*n* = 87, 17.3%), and immune function or fatigue reduction (*n* = 60, 12.0%). Online retail channels were the most prevalent purchase pathway (57.8%), and capsule/tablet formulation was the most frequent format consumed (41.6%).

**Table 1:** Sample characteristics (*N* = 502).

Category	Characteristic	*n*	%
Gender	Female	426	84.9
Gender	Male	76	15.1
Age	20s	126	25.1
Age	30s	277	55.2
Age	40+	99	19.7
Occupation	Employee	345	68.7
Occupation	Student	62	12.4
Occupation	Self-employed	36	7.2
Occupation	Homemaker	30	6.0
Occupation	Professional	29	5.8
Education	Bachelor’s	360	71.7
Education	High school	64	12.7
Education	Associate	49	9.8
Education	Master’s+	29	5.8
Marital status	Single	388	77.3
Marital status	Married	114	22.7
Monthly household income	< KRW 1 M	56	11.2
Monthly household income	KRW 1–2 M	34	6.8
Monthly household income	KRW 2–3 M	152	30.3
Monthly household income	KRW 3–4 M	216	43.0
Monthly household income	≥ KRW 4 M	44	8.8

### 3.2 Measures

#### 3.2.1 Label information cue types

Label information cue types were assessed using a 25-item scale developed through a systematic multi-stage process. In Stage 1, an initial item pool was generated through: (a) a structured literature review on food labeling, functional food health claims, and consumer information processing (Lähteenmäki, 2013; Hung et al., 2017; Szykman et al., 1997); (b) systematic content analysis of 120 commercially available inner beauty supplement product labels; and (c) semi-structured interviews with 12 target consumers. This yielded an initial pool of 42 candidate items across five theoretically derived dimensions. In Stage 2, content validity was assessed by five subject-matter experts using a content validity index (CVI) procedure (Lynn, 1986); items with CVI < 0.80 were revised or eliminated, reducing the pool to 35 items. In Stage 3, a pilot test with 30 inner beauty supplement consumers led to further refinement. The final instrument comprised 25 items (five per subscale: functional emphasis expression, outcome-focused expression, scientific evidence expression, emotional expression, and visual label credibility), rated on a 5-point Likert scale (1 = strongly disagree, 5 = strongly agree).

As a preliminary assessment of dimensionality, exploratory factor analysis (EFA) with principal component analysis (PCA) and Varimax rotation was conducted. The Kaiser-Meyer-Olkin measure of sampling adequacy was 0.955, and Bartlett’s test of sphericity was significant (χ^2^ = 12813.212, df = 300, *p* < 0.001). The exploratory solution suggested a five-factor structure, with total explained variance of 81.47%. Cronbach’s *α* ranged from 0.904 to 0.966 across subscales. The complete wording of all measurement items is provided in Supplementary Table S1.

#### 3.2.2 Consumer trust (product credibility)

Consumer trust was operationalized as perceived product credibility and measured using 8 items. EFA confirmed a single-factor structure (explained variance 85.88%, KMO = 0.957, Bartlett’s χ^2^ = 5539.186, df = 28, *p* < 0.001; α = 0.976). Scale mean: *M* = 3.76 (SD = 1.18).

#### 3.2.3 Purchase intention

Purchase intention was assessed using eight items capturing self-reported purchase likelihood, future purchase plans, label-information-based purchase consideration, and recommendation intention. EFA confirmed a single-factor structure (explained variance 86.92%, KMO = 0.958, Bartlett’s χ^2^ = 5812.115, df = 28, *p* < 0.001; α = 0.978). Scale mean: *M* = 3.79 (SD = 1.21). Skewness values ranged from −1.52 to −1.18 and kurtosis from −0.23 to 3.35, within acceptable normality thresholds (Finch and West, 1997). Measurement properties are summarized in Table 2.

**Table 2:** Measurement properties summary.

Construct	Items	Eigenvalue	Variance (%)	KMO	α	M (SD)
Functional emphasis	5	3.688	14.753	0.955*	0.904	3.94 (0.98)
Outcome-focused	5	4.202	16.809	0.955*	0.944	3.94 (1.01)
Scientific evidence	5	3.862	15.448	0.955*	0.943	4.01 (0.99)
Emotional expression	5	4.321	17.284	0.955*	0.947	3.89 (1.06)
Visual label credibility	5	4.294	17.176	0.955*	0.966	3.83 (1.17)
Overall label cue scale	25	–	81.470	0.955	0.959	3.92 (0.82)
Consumer trust	8	6.870	85.876	0.957	0.976	3.76 (1.18)
Purchase intention	8	6.953	86.918	0.958	0.978	3.79 (1.21)

KMO, Kaiser-Meyer-Olkin; α, Cronbach’s alpha; M, mean; SD, standard deviation. *Combined KMO for the 25-item label cue scale. All Bartlett’s tests significant at *p* < 0.001.

### 3.3 Analytical procedure

Data were analyzed using SPSS Version 28.0, AMOS Version 28.0, and Hayes’ (2013) PROCESS Macro. First, frequency analysis was conducted to describe participants’ sociodemographic characteristics and inner beauty supplement consumption patterns. Second, exploratory factor analysis (EFA) and Cronbach’s alpha were used as preliminary assessments of factor structure and internal consistency. Because the five label-cue dimensions were theoretically related, the EFA results were interpreted as preliminary evidence of dimensionality, whereas the primary evidence for construct validity was provided by confirmatory factor analysis (CFA), average variance extracted (AVE), composite reliability (CR), and the heterotrait–monotrait ratio (HTMT).

Third, to further examine the adequacy of the measurement structure identified through EFA, confirmatory factor analysis (CFA) was conducted using AMOS 28.0. Model fit was evaluated using χ^2^, χ^2^/df, RMR, GFI, AGFI, NFI, TLI, CFI, and RMSEA. Standardized factor loadings, average variance extracted (AVE), and composite reliability (CR) were calculated to assess convergent validity and construct reliability.

Fourth, discriminant validity among the constructs was assessed using the heterotrait–monotrait ratio of correlations (HTMT). HTMT values below 0.85 were considered to indicate adequate discriminant validity. In addition, because all focal variables were measured using self-reported survey responses from the same respondents, common method variance was examined by comparing the baseline measurement model with a common latent factor (CLF) model. The potential influence of common method variance was evaluated by comparing changes in χ^2^/df, RMR, GFI, AGFI, NFI, TLI, CFI, and RMSEA before and after adding the CLF.

After validating the measurement model, descriptive statistics and Pearson correlations were computed for all primary variables. Multiple regression analyses were then conducted to examine the unique associations of the five label cue types with consumer trust and purchase intention. A simple regression was used to examine the association between consumer trust and purchase intention. Variance inflation factors (VIFs) were examined to assess multicollinearity among the predictor variables. Finally, separate bootstrapped indirect association analyses were conducted using Hayes’ PROCESS Model 4 with 5,000 iterations. In each model, one label cue type was entered as the independent variable, consumer trust was specified as the statistical mediator, and purchase intention was entered as the outcome variable. These models were used to estimate the indirect association between each label cue type and purchase intention through consumer trust. Because the five label cue types were not entered simultaneously in these indirect association models, the indirect association results should be interpreted as separate indirect associations rather than as unique indirect associations controlling for all other cue types. All indirect associations were evaluated using 95% bias-corrected confidence intervals. All tests used a two-tailed significance level of *α* = 0.05. Because the five label cue types are conceptually and empirically related, an integrated indirect association model was additionally estimated in which all five cue types were entered simultaneously as predictors of consumer trust and of purchase intention, with consumer trust specified as the statistical mediator. This integrated model allows the relative direct and indirect associations of each cue type to be evaluated within a single framework while controlling for the shared variance among the cue types. Indirect associations in the integrated model were estimated using 5,000 bootstrap resamples with 95% bias-corrected confidence intervals; an interval excluding zero was taken to indicate a statistically significant indirect association.

## 4 Results

### 4.1 Measurement model validation

Before testing the hypotheses, the measurement model was evaluated using confirmatory factor analysis (CFA). The seven-factor measurement model consisted of five label information cue dimensions, consumer trust, and purchase intention. The CFA and construct validity results are summarized in Table 3. The model showed acceptable fit: χ^2^ = 1,473.843, df = 758, χ^2^/df = 1.944, RMR = 0.035, GFI = 0.901, AGFI = 0.878, NFI = 0.944, TLI = 0.970, CFI = 0.972, and RMSEA = 0.043. Although AGFI was slightly below the conventional 0.90 threshold, the remaining fit indices supported an acceptable measurement model.

**Table 3:** Confirmatory factor analysis and construct validity.

Construct	Measurement variable	*B*	*β*	SE	*t*	*p*	AVE	CR
Functional emphasis expression	FEE1	1.000	0.825				0.590	0.878
	FEE2	0.931	0.790	0.046	20.364	***		
	FEE3	0.964	0.797	0.047	20.615	***		
	FEE4	1.005	0.825	0.046	21.670	***		
	FEE5	0.972	0.806	0.046	20.950	***		
Outcome-focused expression	OFE1	1.000	0.902				0.731	0.931
	OFE2	0.957	0.868	0.033	28.770	***		
	OFE3	0.979	0.875	0.033	29.339	***		
	OFE4	0.982	0.867	0.034	28.725	***		
	OFE5	0.994	0.880	0.034	29.667	***		
Scientific evidence expression	SEE1	1.000	0.874				0.731	0.931
	SEE2	0.993	0.871	0.037	27.127	***		
	SEE3	0.999	0.872	0.037	27.201	***		
	SEE4	0.991	0.881	0.036	27.781	***		
	SEE5	0.992	0.882	0.036	27.869	***		
Emotional expression	EE1	1.000	0.894				0.725	0.929
	EE2	0.975	0.877	0.034	29.022	***		
	EE3	0.954	0.863	0.034	27.975	***		
	EE4	0.994	0.891	0.033	30.072	***		
	EE5	1.018	0.896	0.033	30.512	***		
Visual label credibility	VLC1	1.000	0.928				0.787	0.949
	VLC2	0.988	0.919	0.027	37.203	***		
	VLC3	0.971	0.900	0.028	34.832	***		
	VLC4	0.987	0.921	0.026	37.389	***		
	VLC5	0.986	0.943	0.024	40.782	***		
Consumer trust	CT1	1.000	0.935				0.764	0.963
	CT2	0.969	0.914	0.026	37.711	***		
	CT3	0.947	0.915	0.025	37.902	***		
	CT4	0.962	0.908	0.026	36.832	***		
	CT5	0.953	0.900	0.027	35.875	***		
	CT6	0.967	0.910	0.026	37.185	***		
	CT7	0.960	0.926	0.024	39.622	***		
	CT8	0.941	0.918	0.025	38.340	***		
Purchase intention	PI1	1.000	0.949				0.772	0.964
	PI2	0.940	0.906	0.024	38.587	***		
	PI3	0.925	0.921	0.023	41.116	***		
	PI4	0.956	0.920	0.023	40.873	***		
	PI5	0.958	0.915	0.024	39.971	***		
	PI6	0.935	0.923	0.023	41.521	***		
	PI7	0.911	0.914	0.023	39.927	***		
	PI8	0.933	0.929	0.022	42.673	***		

B, unstandardized factor loading; β, standardized factor loading; SE, standard error; AVE, average variance extracted; CR, composite reliability. For model identification, the first loading of each construct was fixed to 1.000. Model fit: χ^2^ = 1,473.843, df = 758, χ^2^/df = 1.944, RMR = 0.035, GFI = 0.901, AGFI = 0.878, NFI = 0.944, TLI = 0.970, CFI = 0.972, RMSEA = 0.043. ***p < 0.001.

All standardized factor loadings ranged from 0.790 to 0.949 and were statistically significant at *p* < 0.001. AVE values ranged from 0.590 to 0.787, exceeding the recommended 0.50 threshold, and CR values ranged from 0.878 to 0.964, exceeding the recommended 0.70 threshold. These results support convergent validity and construct reliability.

Discriminant validity was assessed using the heterotrait–monotrait ratio of correlations (HTMT). The HTMT results are summarized in Table 4. HTMT values ranged from 0.388 to 0.769, and all values were below the conservative 0.85 threshold. The highest HTMT value was observed between consumer trust and purchase intention (0.769), which remained below the threshold. Therefore, the constructs were empirically distinguishable.

**Table 4:** HTMT results for discriminant validity.

Construct	1	2	3	4	5	6	7
1. Functional emphasis expression	–						
2. Outcome-focused expression	0.668	–					
3. Scientific evidence expression	0.711	0.719	–				
4. Emotional expression	0.493	0.389	0.388	–			
5. Visual label credibility	0.521	0.494	0.571	0.626	–		
6. Consumer trust	0.643	0.545	0.579	0.745	0.626	–	
7. Purchase intention	0.766	0.609	0.707	0.608	0.677	0.769	–

HTMT, heterotrait–monotrait ratio of correlations. All HTMT values were below the conservative 0.85 threshold, supporting discriminant validity. *N* = 502.

As an initial diagnostic for common method variance, Harman’s single-factor test indicated that the first unrotated factor accounted for 38.7% of the total variance, below the commonly cited 50% threshold (Podsakoff et al., 2003). However, because Harman’s single-factor test alone is limited as a *post hoc* diagnostic (Richardson et al., 2009), common method variance was further examined by comparing the baseline measurement model with a common latent factor (CLF) model. The model comparison results are summarized in Table 5. The baseline measurement model showed acceptable fit: χ^2^ = 1,473.843, df = 758, χ^2^/df = 1.944, RMR = 0.035, GFI = 0.901, AGFI = 0.878, NFI = 0.944, TLI = 0.970, CFI = 0.972, and RMSEA = 0.043. The CLF model also showed acceptable fit: χ^2^ = 1,298.042, df = 717, χ^2^/df = 1.810, RMR = 0.027, GFI = 0.903, AGFI = 0.880, NFI = 0.951, TLI = 0.974, CFI = 0.977, and RMSEA = 0.040. Although model fit improved slightly after adding the CLF, both models showed acceptable fit and the changes in fit indices were limited. Therefore, common method variance cannot be completely ruled out, but it does not appear to substantially undermine the validity of the measurement model.

**Table 5:** Common latent factor model comparison.

Model	χ^2^	df	χ^2^/df	RMR	GFI	AGFI	NFI	TLI	CFI	RMSEA
Baseline measurement model	1,473.843	758	1.944	0.035	0.901	0.878	0.944	0.970	0.972	0.043
CLF model	1,298.042	717	1.810	0.027	0.903	0.880	0.951	0.974	0.977	0.040

CLF, common latent factor; RMR, root mean square residual; GFI, goodness-of-fit index; AGFI, adjusted goodness-of-fit index; NFI, normed fit index; TLI, Tucker–Lewis index; CFI, comparative fit index; RMSEA, root mean square error of approximation. *N* = 502.

### 4.2 Descriptive statistics and correlations

After confirming the adequacy of the measurement model, descriptive statistics were computed for all primary variables.

Descriptive statistics are presented in Table 6. Scientific evidence expression recorded the highest mean (*M* = 4.01, SD = 0.99), followed by functional emphasis (*M* = 3.94, SD = 0.98) and outcome-focused expression (*M* = 3.94, SD = 1.01). Consumer trust (*M* = 3.76, SD = 1.18) and purchase intention (*M* = 3.79, SD = 1.21) exhibited moderate-to-high mean scores. Skewness and kurtosis values were within Finch and West’s (1997) acceptable thresholds.

**Table 6:** Descriptive statistics (*N* = 502).

Variable	*M*	SD	Min	Max	Skew	Kurt	r (overall)
Label cues overall	3.92	0.82	1.40	5.00	−1.05	0.05	–
Functional emphasis	3.94	0.98	1.00	5.00	−1.20	0.28	0.768***
Outcome-focused	3.94	1.01	1.00	5.00	−1.23	0.06	0.725***
Scientific evidence	4.01	0.99	1.00	5.00	−1.43	0.54	0.739***
Emotional expression	3.89	1.06	1.00	5.00	−1.52	1.04	0.688***
Visual credibility	3.83	1.17	1.00	5.00	−1.31	0.25	0.748***
Consumer trust	3.76	1.18	1.00	5.00	−1.18	−0.23	0.739***
Purchase intention	3.79	1.21	1.00	5.00	−1.28	0.06	0.765***

r = Pearson correlation with overall label cue composite. ***p < 0.001.

Pearson correlation results are presented in Table 7. The overall label cue composite was significantly and positively correlated with consumer trust (*r* = 0.739, *p* < 0.001) and purchase intention (*r* = 0.765, *p* < 0.001). Consumer trust and purchase intention were strongly correlated (*r* = 0.752, *p* < 0.001). Emotional expression exhibited the strongest correlation with consumer trust (*r* = 0.717, *p* < 0.001), while functional emphasis demonstrated the strongest association with purchase intention (*r* = 0.720, *p* < 0.001).

**Table 7:** Pearson correlation matrix (*N* = 502).

Variable	1	2	3	4	5	6	7	8
1. Label cues (overall)	1							
2. Functional emphasis	0.768***	1						
3. Outcome-focused	0.725***	0.618***	1					
4. Scientific evidence	0.739***	0.657***	0.678***	1				
5. Emotional expression	0.688***	0.456***	0.368***	0.367***	1			
6. Visual credibility	0.748***	0.487***	0.472***	0.545***	0.599***	1		
7. Consumer trust	0.739***	0.605***	0.523***	0.556***	0.717***	0.608***	1	
8. Purchase intention	0.765***	0.720***	0.585***	0.679***	0.586***	0.659***	0.752***	1

***p < 0.001.

### 4.3 Associations between label cue types and consumer trust (H1)

Results of the multiple regression predicting consumer trust are presented in Table 8. The model was significant [*F*(5, 496) = 182.681, *p* < 0.001, Adj. *R*^2^ = 0.645], explaining 64.5% of the variance. VIF values (1.664–2.418) were all below 10, confirming absence of multicollinearity. All five label cue types were positively and uniquely associated with consumer trust. Emotional expression showed the strongest standardized association (*β* = 0.482, *p* < 0.001), followed by functional emphasis (*β* = 0.188, *p* < 0.001), scientific evidence expression (*β* = 0.137, *p* < 0.001), visual label credibility (*β* = 0.113, *p* < 0.01), and outcome-focused expression (*β* = 0.084, *p* < 0.05). Hypotheses H1a–H1e were fully supported.

**Table 8:** Multiple regression: Label cue types associated with consumer trust.

Predictor	*B*	SE	*β*	*t*	*p*	VIF	Model fit
(Constant)	−0.681	0.157		−4.347	0.001		Adj. R^2^ = 0.645
Functional emphasis	0.227	0.047	0.188***	4.869	0.001	2.104	*F* = 182.681***
Outcome-focused	0.097	0.045	0.084*	2.177	0.030	2.079	
Scientific evidence	0.162	0.049	0.137***	3.310	0.001	2.418	
Emotional expression	0.534	0.038	0.482***	14.039	0.001	1.664	
Visual credibility	0.114	0.037	0.113**	3.038	0.003	1.947	

*p < 0.05, **p < 0.01, ***p < 0.001. VIF, variance inflation factor. All VIF < 10.

### 4.4 Associations between label cue types and purchase intention (H2)

Results of the multiple regression analysis examining purchase intention are presented in Table 9. The model was significant [*F*(5, 496) = 222.807, *p* < 0.001, Adj. *R*^2^ = 0.689], explaining 68.9% of the variance. Functional emphasis (*β* = 0.346, *p* < 0.001), scientific evidence expression (*β* = 0.232, *p* < 0.001), visual label credibility (*β* = 0.233, *p* < 0.001), and emotional expression (*β* = 0.191, *p* < 0.001) all showed significant unique positive associations with purchase intention. Outcome-focused expression did not show a significant unique association with purchase intention (*β* = 0.034, *p* = 0.348). Accordingly, H2a, H2c, H2d, and H2e were supported, whereas H2b was not supported.

**Table 9:** Multiple regression: label cue types associated with purchase intention.

Predictor	*B*	SE	*β*	*t*	*p*	VIF	Model fit
(Constant)	−0.971	0.151		−6.432	0.001		Adj. *R*^2^ = 0.689
Functional emphasis	0.430	0.045	0.346***	9.578	0.001	2.104	*F* = 222.807***
Outcome-focused	0.041	0.043	0.034 n.s.	0.940	0.348	2.079	
Scientific evidence	0.283	0.047	0.232***	5.985	0.001	2.418	
Emotional expression	0.218	0.037	0.191***	5.935	0.001	1.664	
Visual credibility	0.242	0.036	0.233***	6.705	0.001	1.947	

*p < 0.05, ***p < 0.001. n.s., not significant. All VIF < 10.

### 4.5 Association between consumer trust and purchase intention (H3)

Simple regression analysis showed that consumer trust was positively associated with purchase intention [*β* = 0.752, *p* < 0.001; *F*(1, 500) = 649.996, *p* < 0.001; Adj. *R*^2^ = 0.564]. Hypothesis H3 was supported.

### 4.6 Separate bootstrapped indirect association analyses (H4)

The results of the separate bootstrapped indirect association analyses are summarized in Table 10. In each statistical model, one label cue type was entered as the independent variable, consumer trust was specified as the statistical mediator, and purchase intention was entered as the outcome variable. These terms are used only to describe the statistical model and do not imply causal ordering. The 95% bias-corrected confidence intervals for the indirect associations excluded zero in all five models, indicating statistically significant indirect associations through consumer trust. Accordingly, H4a–H4e were supported in the separate statistical models. However, because the models were estimated separately, these results should not be interpreted as simultaneous or comparative indirect associations among the five label cue types.

**Table 10:** Separate bootstrapped indirect association analysis summary.

Statistical model	Total	Direct	Indirect	SE	LLCI	ULCI	Association pattern
Functional → trust → purchase	0.894	0.519***	0.374***	0.047	0.284	0.472	Direct and indirect association
Outcome → trust → purchase	0.702	0.317***	0.385***	0.046	0.300	0.481	Direct and indirect association
Scientific → trust → purchase	0.828	0.461***	0.367***	0.046	0.282	0.462	Direct and indirect association
Emotional → trust → purchase	0.668	0.110*	0.558***	0.057	0.451	0.675	Direct and indirect association
Visual → trust → purchase	0.684	0.332***	0.352***	0.042	0.274	0.439	Direct and indirect association

*p < 0.05, ***p < 0.001. LLCI/ULCI = lower/upper limits of 95% bias-corrected confidence interval.

#### 4.6.1 Functional emphasis expression

The total association was 0.894. Following inclusion of consumer trust, the direct association remained statistically significant at 0.519 (*p* < 0.001). The indirect association was 0.374 [SE = 0.047, 95% CI (0.284, 0.472)], indicating a significant indirect association through consumer trust, with a remaining significant direct association (H4a supported).

#### 4.6.2 Outcome-focused expression

The total association was 0.702. The indirect association through consumer trust was 0.385 [SE = 0.046, 95% CI (0.300, 0.481)], supporting H4b. The direct association also remained statistically significant in the separate statistical model. Therefore, the result indicates a significant indirect association through consumer trust, with a remaining significant direct association, rather than an exclusive indirect association. This finding should be interpreted cautiously because the separate statistical model does not directly correspond to the full multiple regression model in which all five label cue types were entered simultaneously.

#### 4.6.3 Scientific evidence expression

The total association was 0.828 and the direct association remained statistically significant at 0.461 (*p* < 0.001). The indirect association was 0.367 [SE = 0.046, 95% CI (0.282, 0.462)], indicating a significant indirect association through consumer trust, with a remaining significant direct association (H4c supported).

#### 4.6.4 Emotional expression

Emotional expression showed the largest indirect association through consumer trust [indirect association = 0.558, SE = 0.057, 95% CI (0.451, 0.675)], supporting H4d. The direct association decreased from 0.668 to 0.110 after consumer trust was included in the model but remained statistically significant (*p* = 0.022). Therefore, the result indicates a significant indirect association with a substantial indirect component, while the direct association remained statistically significant.

#### 4.6.5 Visual label credibility

The total association was 0.684 and the direct association remained statistically significant at 0.332 (*p* < 0.001). The indirect association was 0.352 [SE = 0.042, 95% CI (0.274, 0.439)], indicating a significant indirect association through consumer trust, with a remaining significant direct association (H4e supported).

### 4.7 Integrated indirect association model

To complement the separate indirect association analyses, an integrated indirect association model was additionally estimated in which all five label cue types were entered simultaneously as predictors of consumer trust and purchase intention, with consumer trust specified as the statistical mediator. This terminology refers only to the statistical structure of the model and does not imply causal ordering. The consumer trust model explained 64.8% of the variance in consumer trust, and the purchase intention model explained 73.1% of the variance in purchase intention. Consumer trust was significantly associated with purchase intention (*β* = 0.334, *p* < 0.001). The integrated results are summarized in Table 11.

**Table 11:** Integrated indirect association model with all five label cue types entered simultaneously (standardized estimates; bootstrap *N* = 5,000).

Label cue type	Total (β)	Direct (β)	Indirect (β)	95% BC CI (indirect)	Association pattern
Functional emphasis	0.346	0.283***	0.063	[0.022, 0.112]	Direct and indirect association
Outcome-focused	0.034	0.006	0.028	[−0.005, 0.066]	No significant indirect association
Scientific evidence	0.232	0.186***	0.046	[0.009, 0.089]	Direct and indirect association
Emotional	0.191	0.030	0.161	[0.095, 0.234]	Indirect association only in integrated model
Visual label credibility	0.233	0.195***	0.038	[−0.000, 0.086]	No significant indirect association

Standardized estimates from the integrated model with all five label cue types entered simultaneously (*N* = 502). Consumer trust → purchase intention: *β* = 0.334, *p* < 0.001. Consumer trust model *R*^2^ = 0.648; outcome model *R*^2^ = 0.731. Indirect associations and 95% bias-corrected (BC) confidence intervals are based on 5,000 bootstrap resamples; intervals excluding zero indicate a statistically significant indirect association. ***p < 0.001 for the direct association. n.s., not significant.

When all five cue types were entered simultaneously, the pattern of indirect associations differed from that obtained in the separate models. Functional emphasis [indirect *β* = 0.063, 95% CI (0.022, 0.112)] and scientific evidence expression [indirect *β* = 0.046, 95% CI (0.009, 0.089)] retained significant indirect associations through consumer trust while also retaining significant direct associations with purchase intention. Emotional expression showed the largest indirect association [indirect *β* = 0.161, 95% CI (0.095, 0.234)]; its direct association with purchase intention was non-significant in the integrated model (*β* = 0.030, *p* = 0.400), indicating an indirect-association-only pattern in the integrated model. In contrast, the indirect associations of outcome-focused expression [indirect *β* = 0.028, 95% CI (−0.005, 0.066)] and visual label credibility [indirect *β* = 0.038, 95% CI (−0.000, 0.086)] were no longer statistically significant in the integrated model, as their bias-corrected confidence intervals included zero. These results indicate that the significant indirect associations observed for these two cue types in the separate models were attributable in part to shared variance with the other cue types. The divergence between the separate and integrated models underscores that the separate results should be interpreted as cue-specific statistical indirect associations rather than as unique indirect effects, whereas the integrated model identifies the indirect associations that remain robust after controlling for all other cue types.

### 4.8 Hypothesis testing summary

H1a–H1e were supported, indicating that all five label cue types were positively associated with consumer trust. H2a, H2c, H2d, and H2e were supported, whereas H2b was not supported in the multiple regression model predicting purchase intention. Thus, outcome-focused expression was not uniquely associated with purchase intention when the other label cue types were entered simultaneously. H3 was supported, indicating that consumer trust was positively associated with purchase intention. Separate bootstrapped indirect association analyses supported H4a–H4e by showing significant statistical indirect associations between each label cue type and purchase intention through consumer trust. For outcome-focused expression, this result should be interpreted as a significant indirect association in a separate statistical model, not as evidence that outcome-focused expression operates exclusively through consumer trust. However, because the indirect association models were estimated separately, these findings should not be interpreted as evidence of unique indirect effects in a simultaneous indirect association framework.

## 5 Discussion

### 5.1 Theoretical contributions

From a health psychology perspective informed by ELM (Petty and Cacioppo, 1986) and Signaling Theory (Spence, 1973; Erdem and Swait, 1998), this research provides four theoretical contributions.

First, this study develops and tests a theoretically informed five-dimensional taxonomy of inner beauty label information cues. Previous research was mostly focused on single information dimensions such as nutritional labeling, organic labels, or health function claims (Szykman et al., 1997; Grunert and Wills, 2007; Cowburn and Stockley, 2005; Wills et al., 2012). The present taxonomy extends this literature by distinguishing functional emphasis expression, outcome-focused expression, scientific evidence expression, emotional expression, and visual label credibility as empirically separable perceived label cue dimensions. Rather than confirming central- or peripheral-route processing, this taxonomy provides an ELM-informed way to organize label cues according to their informational, evidential, affective, and visual characteristics.

Second, the observed pattern of associations suggests that different perceived label cue types are differentially related to consumer trust and purchase intention. Emotional expression showed the strongest association with consumer trust (*β* = 0.482), whereas functional emphasis showed the strongest unique association with purchase intention (*β* = 0.346). This pattern is broadly consistent with an ELM-informed distinction between information-rich and affective or heuristic cues (Petty and Cacioppo, 1986; Chaiken, 1980). However, because the study did not measure involvement, motivation, ability, cognitive elaboration, argument scrutiny, or health knowledge, these findings should be interpreted as differential associations among perceived label cue dimensions rather than as direct evidence of route-specific processing.

Third, the finding for outcome-focused expression should be interpreted cautiously. In the multiple regression model, outcome-focused expression was not uniquely associated with purchase intention after the other label cue types were entered simultaneously. However, the separate indirect association analysis showed a significant indirect association between outcome-focused expression and purchase intention through consumer trust. From a Signaling Theory perspective, this pattern suggests that result-oriented claims, such as “improved skin radiance after 4 weeks,” may function as credibility-related signals when consumers evaluate product claims under uncertainty (Connelly et al., 2011; Erdem and Swait, 1998). In other words, outcome-focused expressions may be more closely related to purchase intention when they are accompanied by perceived product credibility, but this finding should not be interpreted as evidence that outcome-focused expression operates exclusively through consumer trust.

Fourth, emotional expression showed the largest indirect association through consumer trust, suggesting that affective and aspirational label cues may be closely linked to perceived product credibility in the inner beauty supplement context. However, because the direct association between emotional expression and purchase intention remained statistically significant after consumer trust was included, this result should be interpreted as a significant indirect association with a substantial indirect component, rather than as evidence of a causal process. This finding suggests that emotional expressions may be related to purchase intention both directly and indirectly through consumer trust.

### 5.2 Managerial implications

Because respondents were not exposed to a standardized label stimulus, the following managerial implications should be interpreted as tentative implications based on general label-perception associations rather than as evidence of the effects of specific label designs, brands, price levels, or product formats. First, emotional expression showed the strongest association with consumer trust, indicating that affective and aspirational language may play an important role in perceived product credibility. However, because the direct association between emotional expression and purchase intention remained statistically significant after consumer trust was included, emotional cues should not be viewed solely as trust-building cues. Instead, they may be related to purchase intention both directly and indirectly through consumer trust.

Second, functional emphasis expression showed the strongest unique association with purchase intention among the five cue types (*β* = 0.346). This finding suggests that consumers who perceive labels as providing clear explanations of active ingredient functions may also report stronger purchase intention. From a managerial perspective, inner beauty supplement labels may therefore benefit from presenting specific ingredient–function information in a clear and credible manner, such as explaining how marine collagen peptides are related to dermal elasticity. Such specificity may be perceived as more informative than broad or vague efficacy claims, although these findings should be interpreted as associations rather than as evidence that functional label wording directly increases purchase intention.

Third, the significant indirect association observed for outcome-focused expression suggests that result claims, such as “skin brightening in 8 weeks,” may be more appropriately communicated when accompanied by credibility-supporting information. Rather than assuming that result claims directly generate purchase intention, marketers may consider presenting such claims together with substantiating cues, such as clinical research citations, ingredient concentrations, or certification information, to support perceived product credibility.

Fourth, visual label credibility was positively associated with both consumer trust (*β* = 0.113) and purchase intention (*β* = 0.233), suggesting that professional and coherent label design may be related to favorable consumer evaluations. In practical terms, inner beauty supplement labels may benefit from clear layout, consistent color use, professional visual presentation, and appropriately displayed certification or quality-related marks. However, visual professionalism should not be treated as a substitute for substantiated product information or scientifically justified claims. Rather, visual design elements may support perceived credibility when they are presented accurately, transparently, and in combination with clear functional information and credible evidence (Alberts and Van der Geest, 2011; Liu et al., 2025b).

These findings are also consistent with recent front-of-package labeling research showing that health-related label effects depend on credibility support and design execution. Liu et al. (2025a) reported that health labels may not always increase consumer valuation when presented alone, but that credible explanatory information can reduce skepticism toward health claims. Similarly, Liu et al. (2025b) found that consumers show different preferences across FDA-endorsed “Healthy” label designs, indicating that the visual and informational format of health labels matters. Therefore, inner beauty supplement labels may benefit from combining clear functional information, credible evidence, and visually coherent design elements rather than relying solely on broad health or beauty claims.

Fifth, with 57.8% of the sample purchasing online, the five-cue taxonomy offers a theoretically informed framework for assessing how each cue type may translate from static labels to digital information formats, such as QR code–linked product information (Rotsios et al., 2022). Scientific evidence expressions may lose communicative impact within the space constraints of mobile screens, suggesting information architecture strategies such as progressive disclosure via QR codes or label explainer features.

### 5.3 Limitations and future research

The findings should be interpreted in the context of several limitations. First, the cross-sectional survey design does not permit causal inference. All variables were measured from the same respondents at a single point in time; therefore, the findings should be interpreted as associations rather than causal effects. Future research should employ experimental designs with controlled label stimuli and random assignment, as well as longitudinal panel studies, to examine causal relationships and changes in consumer trust and purchase intention over time.

Second, respondents were not exposed to a standardized label stimulus. Instead, they evaluated label information cues based on inner beauty supplement products that they had purchased or consumed within the preceding 6 months. Consequently, participants may have considered different brands, products, price levels, package designs, label layouts, and product formats when answering the questionnaire. The findings should therefore be interpreted as associations between general label perceptions and purchase intention rather than responses to a single controlled label design.

Third, although additional assessments of common method variance were conducted using both Harman’s single-factor test and a common latent factor (CLF) model, common method bias cannot be completely ruled out because all focal constructs were measured using self-reported Likert-type scales. Future studies should employ procedural remedies such as temporal separation of measurements, multiple data sources, behavioral purchase data, or eye-tracking measures.

Fourth, the study relied on a single South Korean consumer sample, with women accounting for 84.9% of respondents. Therefore, the generalizability of the findings to male consumers, more gender-balanced populations, and consumers in other cultural, market, or regulatory environments may be limited. Future studies should employ more gender-balanced samples and cross-national or cross-cultural designs to examine whether the observed associations are consistent across different consumer groups and market contexts.

Fifth, although the EFA, CFA, and reliability analyses supported the proposed measurement structure, measurement development, measurement validation, and hypothesis testing were conducted using the same dataset. This may raise concerns about sample-specific model fit and potential overfitting. Therefore, the EFA, CFA, AVE, CR, HTMT, and reliability results should be regarded as initial validation evidence rather than as full validation of the newly developed label cue scale. Future research should validate the measurement model using an independent sample or a split-sample approach, in which exploratory analyses are conducted in one subsample and confirmatory analyses and hypothesis testing are conducted in another.

Sixth, several constructs showed high internal consistency coefficients, which may reflect strong coherence among items but may also indicate potential item redundancy. Although the CFA results, AVE and CR values, and HTMT analysis supported convergent validity, construct reliability, and discriminant validity, future studies should further refine the measurement items and validate the scale across independent samples.

Seventh, the primary indirect association analyses were conducted as separate PROCESS Model 4 models. To address the reviewer’s concern regarding shared variance among the five cue types, we additionally estimated an integrated indirect association model in which all five cue types were entered simultaneously. The integrated model showed that the indirect associations of functional emphasis, scientific evidence expression, and emotional expression through consumer trust remained robust after controlling for the other cue types, whereas the indirect associations of outcome-focused expression and visual label credibility were no longer statistically significant. These indirect association results should therefore be interpreted as model-dependent statistical indirect associations rather than as evidence of causal pathways or mechanisms. Nevertheless, because both the separate and integrated models were estimated using the same cross-sectional sample and observed composite variables, future research should validate the indirect association structure using independent samples and latent-variable SEM.

Eighth, the regression and indirect association analyses did not include demographic, consumption-related, or psychographic control variables. Although the sample characteristics and inner beauty supplement consumption patterns were described, variables such as gender, age, income, education, purchase channel, prior product experience, health consciousness, beauty involvement, brand familiarity, and price sensitivity were not included as covariates in the main models. Therefore, the observed associations may partly reflect unmeasured individual differences. Future research should include relevant control variables or conduct robustness checks to examine whether the associations remain stable after accounting for consumer characteristics and prior experience.

## 6 Conclusion

Employing an integrated health psychology perspective informed by ELM and Signaling Theory, this cross-sectional study examined how five distinct label information cue types—functional emphasis expression, outcome-focused expression, scientific evidence expression, emotional expression, and visual label credibility—were associated with consumer trust and self-reported purchase intention among 502 inner beauty supplement consumers. The findings showed that all five label cue types were positively associated with consumer trust, while functional emphasis expression, scientific evidence expression, emotional expression, and visual label credibility were uniquely associated with purchase intention in the multiple regression model.

The separate bootstrapped indirect association analyses showed significant indirect associations between each label cue type and purchase intention through consumer trust. However, the additional integrated indirect association model, in which all five label cue types were entered simultaneously, further clarified that the indirect associations of functional emphasis expression, scientific evidence expression, and emotional expression through consumer trust remained significant after accounting for shared variance among the cue types. In contrast, the indirect associations of outcome-focused expression and visual label credibility were no longer statistically significant in the integrated model. These findings indicate that the indirect association results should be interpreted as model-dependent statistical associations rather than as evidence of causal pathways or mechanisms.

Overall, the findings suggest that consumers may treat certain label cues as credibility-related signals when evaluating health-related supplement products. This trust-related pattern is consistent with health psychology models emphasizing trust in health information processing and health-related decision making (Siegrist et al., 2008). As the inner beauty supplement market expands and regulatory scrutiny of health claims intensifies, the findings suggest that label communication may benefit from combining clear functional information, credible evidence, and visually coherent design rather than relying solely on broad claims or visual appeal.

## Data Availability

The raw data supporting the conclusions of this article will be made available by the authors, without undue reservation.
