# Supplementary material for: Inner beauty supplement label information cues, consumer trust, and purchase intentions: cross-sectional evidence from South Korean consumers
Source: Front Psychol. 2026 Aug 17;17:1864122. doi: 10.3389/fpsyg.2026.1864122 (PMC13526668; doi:10.3389/fpsyg.2026.1864122)
Supplement: Supplementary file 1 [file Table_1.docx]

**Supplementary Table S1 | Measurement Items**

The original questionnaire was administered in Korean. The English translations of the measurement items are presented below. All items were measured on a five-point Likert scale ranging from 1 = strongly disagree to 5 = strongly agree.

| **Construct** | **Item code** | **Item wording** |
| --- | --- | --- |
| Functional emphasis expression | FEE1 | I trust an inner beauty product when its label includes an explanation of skin-related functions. |
| Functional emphasis expression | FEE2 | Phrases focusing on skin function improvement make me expect the product to be effective. |
| Functional emphasis expression | FEE3 | Functional expressions such as “may help the skin” influence my product choice. |
| Functional emphasis expression | FEE4 | Phrases emphasizing functionality are perceived as communicating the product’s efficacy more clearly. |
| Functional emphasis expression | FEE5 | I prefer products that mention functionality over products without functional expressions. |
| Outcome-focused expression | OFE1 | Specific outcome expressions such as “skin improved after four weeks of use” increase my trust. |
| Outcome-focused expression | OFE2 | I trust a product more when I see numerical changes in users’ skin on the label. |
| Outcome-focused expression | OFE3 | Labels that express skin improvement effects numerically have a positive influence on my purchase. |
| Outcome-focused expression | OFE4 | Visible outcome figures on the label increase my expectations for the product. |
| Outcome-focused expression | OFE5 | The more specific the skin outcome expression is, the more likely I am to choose the product. |
| Scientific evidence expression | SEE1 | Scientific information such as “clinically tested” or “1,000 mg collagen peptides” increases my trust. |
| Scientific evidence expression | SEE2 | I trust a product more when experimental evidence is specified on the label. |
| Scientific evidence expression | SEE3 | I trust a product more when the label includes explanations based on papers or data. |
| Scientific evidence expression | SEE4 | Explanations based on scientific figures show the product’s professionalism. |
| Scientific evidence expression | SEE5 | Scientific evidence expressions in inner beauty products give me confidence in their effects. |
| Emotional expression | EE1 | Emotional expressions such as “vitality for the skin” or “elastic skin” feel attractive. |
| Emotional expression | EE2 | Emotional phrases induce a positive perception of the product. |
| Emotional expression | EE3 | Sensory image expressions increase my expectations of the product’s quality. |
| Emotional expression | EE4 | Products containing emotional phrases feel softer and more effective. |
| Emotional expression | EE5 | Emotion-focused label phrases elicit emotional trust. |
| Visual label credibility | VLC1 | I trust a product when visual elements such as color, design, and icons on the label look professional. |
| Visual label credibility | VLC2 | A clean and consistent label design increases product credibility. |
| Visual label credibility | VLC3 | Visual elements including official marks or certification marks induce trust. |
| Visual label credibility | VLC4 | A design that conveys professionalism creates a positive impression of the product. |
| Visual label credibility | VLC5 | When package design looks premium, I tend to trust the product’s functionality as well. |
| Consumer trust (perceived product credibility) | CT1 | I tend to trust the label information of the inner beauty product. |
| Consumer trust (perceived product credibility) | CT2 | I feel that the functional expressions of the inner beauty product are accurate. |
| Consumer trust (perceived product credibility) | CT3 | I think the quality of the inner beauty product is excellent. |
| Consumer trust (perceived product credibility) | CT4 | The product’s ingredients and efficacy are displayed in a trustworthy manner. |
| Consumer trust (perceived product credibility) | CT5 | I tend to trust surrounding evaluations or reviews of the inner beauty product. |
| Consumer trust (perceived product credibility) | CT6 | The scientific evidence presented on the product label gives me trust. |
| Consumer trust (perceived product credibility) | CT7 | I have a high level of trust in the inner beauty product brand. |
| Consumer trust (perceived product credibility) | CT8 | I think I would not regret choosing this product. |
| Purchase intention | PI1 | I am likely to actually purchase an inner beauty product. |
| Purchase intention | PI2 | I would like to purchase an inner beauty product if it seems effective for skin improvement. |
| Purchase intention | PI3 | I consider purchasing a product when the expressions on the inner beauty label give me trust. |
| Purchase intention | PI4 | I plan to purchase inner beauty products that emphasize skin functionality in the future. |
| Purchase intention | PI5 | I have made a purchase decision based on the label of an inner beauty product. |
| Purchase intention | PI6 | I want to purchase products with impressive skin-related expressions. |
| Purchase intention | PI7 | I will purchase an inner beauty product if I perceive it to have high quality or credibility. |
| Purchase intention | PI8 | When choosing an inner beauty product, I make decisions by referring to the information on the label. |

*Note. FEE = functional emphasis expression; OFE = outcome-focused expression; SEE = scientific evidence expression; EE = emotional expression; VLC = visual label credibility; CT = consumer trust; PI = purchase intention.*
